# Relationships Among Pre-Pregnancy BMI, Gestational, and Postpartum Oral Glucose Tolerance Results in Women With Gestational Diabetes Mellitus

**DOI:** 10.3389/fnut.2021.714690

**Published:** 2021-12-01

**Authors:** Chunmei Mi, Hong Liu, Hongying Peng, Chunxia Cheng, Meng Wang, Hua Liu, Guo Feng, Jinru Wu, Hao Nie, Min Liu

**Affiliations:** ^1^Department of Obstetrics and Gynecology, The Third Xiangya Hospital, Central South University, Changsha, China; ^2^Department of Nutrition, The Third Xiangya Hospital, Central South University, Changsha, China; ^3^Department of Central Sterile Supply, The Third Xiangya Hospital, Central South University, Changsha, China; ^4^Department of Geriatrics, The First Affiliated Hospital of Hunan Normal University, Changsha, China

**Keywords:** gestational diabetes mellitus, medical nutrition therapy, BMI, postpartum glucose tolerance, diabetes mellitus

## Abstract

**Background and Aims:** To investigate the relationship among maternal demographic and clinical characteristics, gestational and postpartum oral glucose tolerance test (ppOGTT) results in patients with gestational diabetes mellitus (GDM).

**Methods:** Patients with gestational diabetes mellitus from January 1, 2016, to August 31, 2019, were enrolled. General characteristics, dietary energy intake, pre-gestational body mass index (BMI), gestational oral glucose tolerance test (gOGTT), and 42 days ppOGTT results of all participants were collected. The relationships among maternal clinical characteristics, fasting glucose of gOGTT (gOGTT-FPG), 1 h postprandial glucose of gOGTT (gOGTT-1h PG), 2 h postprandial glucose of gOGTT (gOGTT-2h PG), and maternal postpartum glucose outcomes were evaluated.

**Results:** A total of 156 patients with GDM were included in this study. Among them, 73.7% had inadequate daily total energy intake, an insufficient ratio of carbohydrates and protein, and an excessive fat ratio. Most of the patients (81.4%) were normal in their ppOGTT examination. Less than 20% of the patients (16.7%) were in the pre-diabetic situation, and 3 patients (1.9%) had diabetes. Pre-pregnancy BMI of patients with GDM was a risk factor for increased gOGTT-FPG levels. Those who were overweight before pregnancy had a greater risk for a higher gOGTT-FPG compared to those who had normal pre-pregnancy BMI (*P* = 0.021*, odds ratio* [*OR*] = 4.583). Abnormal gOGTT-2hPG was a risk factor for abnormal ppOGTT (*P* = 0.04). Those who had an elevated gOGTT-2hPG (≧8.5 mmol/L) had a 2.426 times higher risk for abnormal ppOGTT than those who had normal gOGTT-2hPG (<8.5 mmol/L) results.

**Conclusion:** For women who are overweight before pregnancy, it is better to control their BMI to normal before getting pregnant. Women who had abnormal gOGTT-2h PG should pay more attention to the ppOGTT results.

## Introduction

Gestational diabetes mellitus (GDM) is defined as “glucose intolerance with onset or first recognition during pregnancy” or “carbohydrate intolerance of varying severity which is diagnosed in pregnancy and may or may not resolve after pregnancy” (1). GDM is associated with adverse maternal, fetal, and/or neonatal outcomes, such as pregnancy-induced hypertension (PIH) and subsequent cesarean section, premature birth, miscarriage, macrosomia, and respiratory distress syndrome (RDS) (2). Most notably, women with a history of GDM have more than a 7-fold increased risk of developing impaired glucose tolerance (IGT) or type 2 diabetes within 5–10 years following pregnancy (3). Furthermore, children born to mothers with GDM are more likely to develop type 2 diabetes later in life (3). It plays an important role in the prevention and treatment of GDM to identify the susceptible causes, find the factors that may indicate the poor outcomes, and carry out appropriate interventions.

International Association of Diabetes and Pregnancy Study Group (IADPSG) criteria were published and modified by the Ministry of Health (MOH) in China in August 2014 (4, 5), which has proven appropriate for the Chinese population (6). By the new criteria, pregnant women are identified as GDM between 24 and 28 weeks of gestation. GDM is diagnosed if one or more of the following criteria are met after the 75 g gestational oral glucose tolerance test (gOGTT): a fasting plasma glucose (gOGTT-FPG) level ≥ 5.1 mmol/L, a 1 h postprandial glucose (gOGTT-1h PG) level ≥ 10.0 mmol/L, and/or a 2-h postprandial glucose (gOGTT-2h PG) level ≥ 8.5 mmol/L (7).

Previous studies have reported that high-total fat and cholesterol and overall high-energy intake are significant risk factors for GDM, with total fat intake found to be significantly higher among women with GDM than in pregnant women who do not develop GDM (5–7). Several studies had found that pre-pregnancy BMI or gestational BMI was predictive for GDM (8, 9). We aimed to evaluate the specific relationship between demographic and clinical characteristics and the time point of gOGTT (gOGTT-FPG, gOGTT-1h PG, or gOGTT-2h PG). Moreover, we aimed to find which parameter, gOGTT-FPG, gOGTT-1h PG, or gOGTT-2h PG, has the greatest impact on postpartum glucose abnormality.

## Materials and Methods

### Study Design and Participants

We recruited patients diagnosed with GDM during the 24–28 weeks of pregnancy. Inclusion criteria were diagnosis of GDM according to the IADPSG criteria (5) and ability to give informed consent. Exclusion criteria were diagnosis of diabetes before pregnancy, twins or multiple births, and patients who were taking medications known to influence glucose homeostasis before enrollment. This study was approved by the Institutional Review Boards of the Third Xiangya Hospital, Central South University (no. 2020S262). Written informed consent was obtained from all participants before entering the trial.

### Procedures

Gestational diabetes mellitus patients from January 1, 2016, to August 31, 2019, were enrolled. As part of interviewer-administered questionnaires at recruitment, information of age, nationality, educational level, pre-pregnancy weight, family history of diabetes, history of abnormal pregnancy, maternal history of GDM in previous pregnancies, gOGTT-FPG, gOGTT-1h PG, and gOGTT-2h PG were collected. The glucose values were obtained from medical records. And then, they underwent a dietary survey at baseline about 24-h food records by two dieticians, such as daily energy, protein, carbohydrate, and fat intake. Various portion sizes demonstrated to use standardized household measuring utensils, and pictures of food were used to help patients accurately quantify their food and beverage intake. Nutrient analysis software (Shanghai Zhen Ding Health Science Technology Co. Ltd., Shanghai, China) combined with a database composed of locally available foods were used to calculate the total daily energy intake for each patient (10–13). And we estimated adequate or inadequate nutrient intake based on the Chinese Dietary Reference Intake (DRI) 2013 (14). The cutoff values of inadequate and excessive carbohydrate intake are 117 and 143 g, respectively. The cutoff values of inadequate and excessive protein intake are 63 and 77 g, respectively. The cutoff values of inadequate and excessive fat intake are 20 and 30%, respectively.

Pre-pregnancy BMI was categorized as underweight, normal weight, and overweight group (<18.5, 18.5–23.9, and ≥24 kg/m^2^, respectively), according to the China National Diabetes and Metabolic Disorders Study Group, 2002 (10–13). BMI was defined as weight/height squared (kg/m^2^). Maternal height (to the nearest 0.1 cm) and weight (to 0.01 kg) were measured by trained research staff using a stadiometer (SECA model 213, Hamburg, Germany) and a weighing scale (SECA model 803), respectively, with outer garments and shoes removed. Gestational weight gain during pregnancy was calculated by subtracting self-reported pre-pregnancy weight from the weight measured before delivery. Pre-pregnancy BMI and weeks of pregnancy were used to calculate the daily recommended energy intake for each patient with GDM, according to the Dietary Guide for Patients with GDM released in 2018 by the National Health Commission of China (WS/T 601-2018) (15, 16). Pre-pregnancy BMI was <18.5 kg/m^2^, recommended average energy intake (kcal/d) was 2,000–2,300; pre-pregnancy BMI was 18.5–23.9 kg/m^2^, recommended average energy intake (kcal/d) was 1,800–2,100; and pre-pregnancy BMI ≥ 24 kg/m^2^,recommended average energy intake (kcal/d) was 1,500–1,800. Patients with GDM were followed up every 2–4 weeks, and the nutritionist provided dietary adjustment guidance based on blood glucose and dietary survey results. All participants checked their self-monitored blood glucose results and physical exercise status on their cell phone applications, namely, WeChat (the most widely used platform among the Chinese population), to monitor the blood glucose levels of the participants and control the weight gain of patients regularly. Subjects visited the nutrition department every 1–2 weeks. Those who were on regular follow-up and finished 42 days postpartum OGTT were included in our final study. A 75 g oral glucose test was performed 42 days after delivery following GDM. The American Diabetes Association guidelines in 2016 were used to define the postpartum blood glucose outcomes (15, 16). Three situations were recorded: normal glucose range (NGR), fasting glucose <6.1 mmol/L and 2 h glucose <7.8 mmol/L; pre-diabetic situation, impaired fasting glucose (IFG), 6.1 mmol/L ≦fasting blood glucose <7.0 mmol/L and 2 h glucose <7.8 mmol/L or IGT, fasting glucose <6.1 mmol/L, and 7.8 mmol/L ≤ 2 h glucose <11.1 mmol/L; and diabetes mellitus (DM), fasting glucose ≥7.0 mmol/L or 2 h glucose ≥11.1 mmol/L. All participants were diagnosed with GDM at 24–28 weeks of pregnancy. And the gOGTT-FPG, OGTT-1hPG, and OGTT-2hPG were those performed at diagnosis. GDM patients' gOGTT-FPG <5.1 mmol/L was defined as A1 group, gOGTT-FPG ≧5.1 mmol/L was defined as A2 group; gOGTT-1hPG <10.0 mmol/L was defined as B1 group, gOGTT-1hPG ≥ 10.0 mmol/L was defined as B2 group; gOGTT-2hPG <8.5 mmol/L was defined as C1 group, and gOGTT-2hPG ≥ 8.5 mmol/L was defined as C2 group. Further logistic analysis was used to explore predictors for abnormal gOGTT-FPG, gOGTT-1hPG, gOGTT-2hPG, and abnormal postpartum glucose results following GDM.

### Statistical Methods

This study used Epidata software for data entry and SPSS 17.0 statistical software for analysis. Normal distributions were expressed as means with SDs ($\bar{x}$ ± s). A chi-square test and Fisher's exact test were used to compare the count data. Pearson correlation analysis was used to analyze the correlation between two bivariate normal distributions. Spearman correlation analysis was used for analyzing non-bivariate normal distributions. We included demographic and clinical parameters as independent variables and gOGTT-FPG, gOGTT-1hPG, gOGTT-2hPG (or postpartum OGTT results) as dependent variables into the stepwise logistic regression model. A *P* < 0.05 was considered statistically significant.

## Results

### General Characteristics, Maternal, and Newborn Outcomes of the Participants

**Figure 1:**
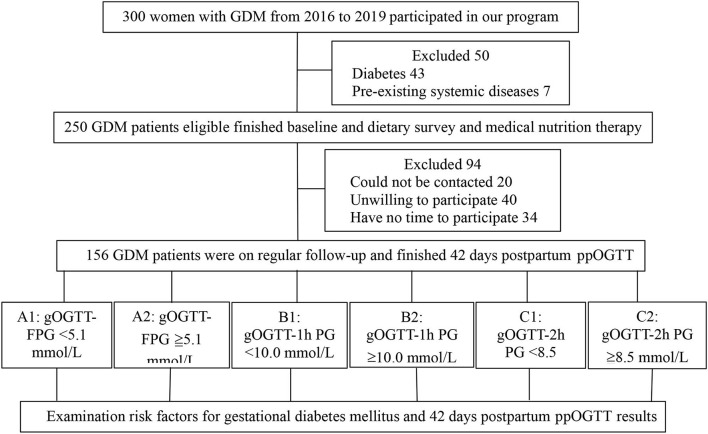
Participants flow chart. GDM, gestational diabetes mellitus; gOGTT, gestational oral glucose tolerance test; FPG, fasting plasma glucose; 1h, 1 hour; 2h, 2 hours; PG, postprandial glucose; IFG, impaired fasting glucose; IGT, impaired glucose tolerance; DM, diabetes mellitus; ppOGTT, postpartum oral glucose tolerance test.

A total of 300 pregnant women with GDM were included in the initial sample. After excluding 50 women, 43 women with pre-gestational or overt diabetes, and 7 pre-existing systemic diseases, a total of 250 patients finished baseline and dietary survey and medical nutrition therapy (Figure 1). Finally, 156 patients finished the follow-up and postpartum glucose re-examination, their general characteristics, maternal and newborn outcomes are given in Table 1. There were 51 cases of GDM women with natural births, which accounts for 34.0% and 103 cases of cesarean section births, which accounts for 66.0%. There were 10 cases of newborns with macrosomia, accounting for 6.4%. The results of 42 postpartum screening indicate that 127 patients with GDM cases had normal blood glucose levels (81.4%), 29 cases were pre-diabetic or diabetes (18.6%), 25 cases had IGT (16.0%), 1 case had IFG (0.6%), and 3 cases had DM (1.9%) (Table 1).

**Table 1:** General characteristics, maternal and newborn outcomes of the participants (*n* = 156).

**Characteristics**	**n/total (%)** **Mean ± SD**
**Race**	
Han nationality	150/156 (96.2)
Other	6/156 (3.8)
**Education level**	
High school degree or lower	46/156 (29.5)
College or bachelor's degree	98/156 (62.8)
Master's degree or above	12/156 (7.7)
**Parity**	
Primipara	84/156 (53.8)
Multipara	72/156 (46.2)
**History of miscarriage**	
Yes	68/156 (43.6)
No	88/156 (56.4)
**History of macrosomia**	
Yes	5/156 (3.2)
No	151/156 (96.8)
**History of GDM**	
Yes	10/156 (6.4)
No	146/156 (93.6)
**Delivery mode**	
Natural births (%)	51/156 (34)
Cesarean section (%)	103/156 (66)
**Macrosomia**	
Yes (%)	10/156 (6.4)
No (%)	146/156 (93.5)
**Maternal complications**	
Yes (%)	8/156 (5.1)
No (%)	148/156 (94.9)
**Healthy neonate**	
Yes (%)	143/156 (91.7)
NICU (%)	13/156 (8.3)
**42-day ppOGTT**	
Normal	127/156 (81.4)
IGT	25/156 (16.0)
IFG	1/156 (0.6)
DM	3/156 (1.9)
Age (years)	31.05 ± 3.87 (24.0~42.0)
<35	124/156 (79.5)
≥35	32/156 (20.5)
Pre-pregnancy weight (kg)	55.29 ± 7.64 (38.0~81.0)
Pre-pregnancy BMI (kg/m^2^)	21.81 ± 2.70 (16.01~30.49)
Underweight	17/156 (10.9)
Normal weight	111/156
Overweight	28/156 (17.9)
Weight at delivery (kg)	68.00 ± 8.35 (50.0~102.0)
BMI at delivery (kg/m^2^)	26.85 ± 3.08 (20.8~36.1)
Weight gain during pregnancy (kg)	55.29 ± 7.64 (−8.5~28.0)
gOGTT-FPG	4.80 ± 0.68 (3.2~7.2)
gOGTT-1hPG	9.84 ± 1.55 (6.0~14.0)
gOGTT-2hPG	8.39 ± 1.50 (4.4~12.9)
ppOGTT-FPG	4.92 ± 0.52 (3.9~6.2)
ppOGTT-1hPG	8.47 ± 1.74 (4.4~14.9)
ppOGTT-2hPG	6.71 ± 1.55 (3.6~13.6)
HbA1C (%)	5.3 ± 1.40
**Family history of diabetes**	
Yes	35/156 (22.4)
No	121/156 (77.6)

*GDM, gestational diabetes mellitus; SD, standard deviation; BMI, body mass index; gOGTT, gestational oral glucose tolerance test; FPG, fasting plasma glucose; 1h, 1 hour; 2h, 2 hours; PG, postprandial glucose; NICU, neonatal intensive care unit; IFG, impaired fasting glucose; IGT, impaired glucose tolerance; DM, diabetes mellitus; ppOGTT, postpartum oral glucose tolerance test*.

### Dietary Survey Results of Participants

Patients with GDM nutrient intake results were recorded upon the first visit to the nutrition department (Table 2). As shown in Table 2, only 3.9% of patients had insufficient carbohydrate intake, 7.7% had normal carbohydrate intake, and 88.4% had excessive carbohydrate intake. Most (60.3%) patients had insufficient protein intake, few (19.2%) had normal protein intake, and 20.5% had excessive protein intake. No one had an insufficient fat intake, only 0.6% had a normal fat intake, and 99.4% had excessive fat intake. Most (73.7%) patients with GDM had insufficient energy intake, few (18.6%) had normal energy intake, while only 7.7% had excessive energy intake. Patients who had normal carbohydrate rates were 73 (46.8%), normal protein rate was 65 (41.7%), and normal fat rate was 30 (19.2%).

**Table 2:** Summary of nutrient intake data of GDM patients recorded at the first visit to the nutrition department (*n* = 156).

	**Nutrient intake**	**n/total (%)**
**Carbohydrate**		
Insufficient	<117 g	6/156 (3.9)
Normal	117–143 g	12/156 (7.7)
Excessive	>143 g	138/156 (88.4)
**Protein**		
Insufficient	<63 g	94/156 (60.3)
Normal	63–77 g	30/156 (19.2)
Excessive	>77 g	32/156 (20.5)
**Fat**		
Insufficient	<25 g	0/156 (0.0)
Normal	25–30 g	1/156 (0.6)
Excessive	>30 g	155/156 (99.4)
**Energy**		
Insufficient	<1,890 kcal	115/156 (73.7)
Normal	1,890–2,310 kcal	29/156 (18.6)
Excessive	>2310 kcal	12/156 (7.7)
**Contribute rate of carbohydrate**		
Insufficient	<45%	27/156 (17.3)
Normal	45–55%	73/156 (46.8)
Excessive	>55%	56/156 (35.9)
**Contribute rate of protein**		
Insufficient	<15%	83/156 (53.2)
Normal	15–20%	65/156 (41.7)
Excessive	>20%	8/156 (5.1)
**Contribute rate of fat**		
Insufficient	<25%	6/156 (3.9)
Normal	25–30%	30/156 (19.2)
Excessive	>30%	120/156 (76.9)

*GDM, gestational diabetes mellitus*.

### Relationship Among Pre-Pregnancy BMI and gOGTT Results

There were no statistically significant differences between groups A1 vs. A2, B1 vs. B2, and C1 vs. C2 regarding parameters, such as age, race, education level, number of total pregnancies, parity, history of miscarriages, history of macrosomia, history of GDM, family history of diabetes, and some outcomes (delivery mode, macrosomia, maternal complications, or neonatal health). Single-factor analysis indicated that patients in group A2 (gOGTT-FPG ≧5.1 mmol/L) had higher pre-pregnancy BMI (22.81 ± 3.20 vs. 21.16 ± 2.08, *P* = 0.001) and higher BMI at delivery (kg/m^2^) (27.67 ± 3.30 vs. 26.31 ± 2.81, *P* = 0.006) than patients in group A1 (gOGTT-FPG <5.1 mmol/L), and this difference was statistically significant. However, there was no significant difference in weight gain during pregnancy between the two groups (12.96 ± 6.01 vs. 12.32 ± 5.52, *P* = 0.501). There was no significant difference in pre-pregnancy BMI (kg/m^2^) (21.62 ± 2.73 vs. 21.99 ± 2.68, *P* = 0.39, 22.02 ± 2.91 vs. 21.65 ± 2.53, *P* = 0.393), between groups B1 and B2, groups C1 and C2 (Table 3).

**Table 3:** Comparisons of various characteristics according to OGTT classification.

	**Group A1**	**Group A2**	***P***	**Group B1**	**Group B2**	***P***	**Group C1**	**Group C2**	***P***
	**[n (%)]**	**[n (%)]**		**[n (%)]**	**[n (%)]**		**[n (%)]**	**[n (%)]**	
**Age (years)**									
<35	78(83.0)	46(74.2)	0.184	64 (85.3)	60 (74.1)	0.082	56 (81.2)	68 (78.2)	0.645
≥35	16(17.0)	16(25.8)		11 (14.7)	21 (25.9)		13 (18.8)	19 (21.8)	
**Race**									
Han nationality	89(94.7)	61(98.4)	0.403	72 (96.0)	78 (96.3)	1.000	68 (98.6)	1 (1.4)	0.229
Other	5 (5.3)	1 (1.6)		3 (4.0)	3 (3.7)		82 (94.3)	5 (5.7)	
**Education level**									
High school degree or lower	27 (28.7)	19 (30.6)	0.900	28 (37.3)	18 (22.2)	0.117	18 (26.1)	28 (32.2)	0.446
College or bachelor's degree	59 (62.8)	39 (62.9)		42 (56.0)	56 (69.1)		47 (68.1)	51 (58.6)	
Master's degree or above	8 (8.5)	4 (6.5)		5 (6.7)	7 (8.6)		4 (5.8)	8 (9.2)	
**Number of total pregnancies**									
<3(%)	68 (72.3)	42 (67.7)	0.538	50 (66.7)	60 (74.1)	0.311	53 (76.8)	57 (65.5)	0.124
≥3 (%)	26 (27.7)	20 (32.3)		25 (33.3)	21 (25.9)		16 (23.2)	30 (34.5)	
**Parity**									
Primipara (%)	56 (59.6)	28 (45.2)	0.077	40 (53.3)	43 (53.1)	0.975	40 (58.0)	44 (50.6)	
Multipara (%)	38 (40.4)	34 (54.8)		35 (46.7)	38 (46.9)		29 (42.0)	43 (49.4)	0.357
**History of miscarriage**									
Yes (%)	39 (41.5)	29 (46.8)	0.515	39 (41.5)	29 (46.8)	0.515	25 (36.2)	43 (49.4)	0.099
No (%)	55 (58.5)	33 (53.2)		55 (58.5)	33 (53.2)		44 (63.8)	44 (50.6)	
**History of macrosomia**									
Yes (%)	3 (3.2)	2 (3.2)	1.000	1 (1.3)	4 (4.9)	0.369	0 (0.0)	5 (5.7)	0.067
No (%)	91 (96.8)	60 (96.8)		74 (98.7)	77 (95.1)		69 (100.0)	82 (94.3)	
**History of GDM**									
Yes (%)	6 (6.4)	4 (93.6)	1.000	5 (6.7)	5 (6.2)	1.000	2 (2.9)	8 (9.2)	0.187
No (%)	88 (93.6)	58 (93.5)		70 (93.3)	76 (93.8)		67 (97.1)	79 (90.8)	
**Family history of diabetes**									
Yes (%)	18 (19.1)	17 (27.4)	0.226	14 (18.7)	21 (25.9)	0.278	13 (18.8)	22 (25.3)	0.338
No (%)	76 (80.9)	45 (72.6)		61 (81.3)	60 (74.1)		56 (81.2)	65 (74.7)	
Pre-pregnancy weight (kg)*	53.50 ± 6.14	50.02 ± 8.85	**0.001**	54.68 ± 7.43	55.86 ± 7.83	0.334	56.45 ± 8.90	54.38 ± 6.37	0.105
Pre-pregnancy BMI (kg/m^2^)*	21.16 ± 2.08	22.81 ± 3.20	**0.001**	21.62 ± 2.73	21.99 ± 2.68	0.390	22.02 ± 2.91	21.65 ± 2.53	0.393
Weight at delivery (kg)*	66.46 ± 7.50	70.34 ± 9.06	**0.004**	68.19 ± 7.63	67.82 ± 9.90	0.782	69.24 ± 9.56	67.02 ± 7.15	0.099
BMI at delivery (kg/m^2^)*	26.31 ± 2.81	27.67 ± 3.30	**0.006**	27.00 ± 3.03	26.71 ± 3.13	0.561	27.06 ± 3.31	26.69 ± 2.90	0.455
Weight gain during pregnancy (kg)	12.96 ± 6.01	12.32 ± 5.52	0.501	13.51 ± 2.50	11.96 ± 3.10	0.120			
Insufficient	31 (33.0)	21 (33.9)	0.632	19 (25.3)	33 (40.7)		26 (37.7)	26 (29.9)	0.315
Normal	34 (36.2)	26 (41.9)		33 (44.0)	27 (33.3)		22 (31.9)	38 (43.7)	
Excessive	29 (30.9)	15 (24.2)		23 (30.7)	21 (25.9)		21 (30.4)	23 (26.4)	
gOGTT-FPG*	4.36 ± 0.41	5.46 ± 0.43	** <0.001**	4.73 ± 0.64	4.86 ± 0.71	0.244	4.97 ± 0.53	4.66 ± 0.76	**0.003**
gOGTT-1hPG*	9.91 ± 1.16	9.73 ± 2.01	0.542	8.58 ± 1.01	11.00 ± 0.942	** <0.001**	9.41 ± 1.55	10.18 ± 1.48	**0.002**
gOGTT-2hPG*	8.54 ± 1.21	8.16 ± 0.58	0.156	8.14 ± 1.28	8.62 ± 1.66	**0.043**	7.09 ± 0.98	9.41 ± 0.94	** <0.001**
**42-day ppOGTT screening**									
Normal*	78 (83.0)	49 (79.0)	0.691	62 (82.7)	65 (80.2)	0.936	61 (88.4)	66 (75.9)	**0.046**
IFG+IGT	15(16.0)	11 (17.8)		12 (16.0)	14 (17.3)		7 (10.1)	19 (21.8)	
DM	1 (1.0)	2 (3.2)		1 (1.3)	2 (2.5)		1 (1.5)	2 (2.3)	
**Delivery mode**									
Natural birth	32 (34.0)	21 (33.9)	0.982	28 (37.3)	25 (30.9)	0.394	28 (40.6)	25 (28.7)	0.121
Cesarean section	62 (66.0)	41 (66.1)		47 (62.7)	56 (69.1)		41 (59.4)	62 (71.3)	
**Macrosomia**									
Yes	5 (5.3)	7 (11.3)	0.222	5 (5.3)	7 (11.3)	0.222	6 (8.7)	6 (6.9)	0.675
No	89 (94.7)	55 (88.7)		89 (94.7)	55 (88.7)		63 (91.3)	81 (93.1)	
**Maternal complications**									
Yes	5 (5.3)	3 (4.8)	1.000	4 (5.3)	4 (4.9)	1.000	6 (8.7)	2 (2.3)	0.140
No	89 (94.7)	59 (95.2)		71 (94.7)	77 (95.1)		63 (91.3)	85 (97.7)	
**Healthy neonate**									
Yes	86 (91.5)	57 (91.9)	0.921	69 (92.0)	74 (91.4)	0.885	62 (89.9)	81 (93.1)	0.466
NICU	8 (8.5)	5 (8.5)		6 (8.0)	7 (8.6)		7 (10.1)	6 (6.9)	

*GDM, gestational diabetes mellitus; SD, standard deviation; BMI, body mass index; gOGTT, gestational oral glucose tolerance test; FPG, fasting plasma glucose; 1h, 1 hour; 2h, 2 hours; PG, postprandial glucose; NICU, neonatal intensive care unit; ppOGTT, postpartum oral glucose tolerance test; IFG, impaired fasting glucose; IGT, impaired glucose tolerance.*

gOGTT-FPG <5.1 mmol/L was defined as A1 group, gOGTT-FPG ≧5.1 mmol/L was defined as A2 group; gOGTT-1hPG <10.0 mmol/L was defined as B1 group, gOGTT-1hPG ≧ 10.0 mmol/L was defined as B2 group; gOGTT-2hPG <8.5 mmol/L was defined as C1 group, gOGTT-2hPG ≧8.5 mmol/L was defined as C2 group. The bold values means p < 0.05 and the difference was statistically significant. ^*^ means p < 0.05 and the difference was statistically significant.

When gOGTT-FPG was taken as the dependent variable, age, ethnicity, education level, level of physical activity, menstrual regularity, history of macrosomia, history of GDM, family history of diabetes, history of gestational hypertension, number of pregnancy and delivery, parity, and pre-pregnancy BMI were taken as the independent variables, only pre-pregnancy BMI entered into the equation in stepwise logistic analysis. The results showed that pre-pregnancy BMI is a risk factor for increased gOGTT-FPG levels, and the risk of elevated gOGTT-FPG in patients with GDM who were overweight before pregnancy was 4.583 times higher than that of patients with normal weight (*P* = 0.021, Table 4). Pre-pregnancy BMI is not a risk factor for increased gOGTT-1h PG or gOGTT-2h PG levels (gOGTT-1h PG or gOGTT-2h PG as a dependent variable, respectively, the above variables as independent variables, no variables were entered into the equation in logistic regression analysis).

**Table 4:** The relationship between pre-pregnancy BMI and gOGTT-FPG levels (*n* = 156).

	***b***	***S_**b**_***	***Wald x^**2**^***	***P***	***EXP(b)***
Constant	−0.606	0.508	1.426	0.232	0.545
**Pre-pregnancy BMI**					
Normal weight	–	—	12.721	0.002	—
underweight	−0.128	0.547	0.055	0.815	0.880
Overweight*	1.522	0.508	1.426	**0.021**	**4.583**

GDM, gestational diabetes mellitus; BMI, body mass index; gOGTT, gestational oral glucose tolerance test; FPG, fasting plasma glucose. The bold values means p < 0.05 and the difference was statistically significant. ^*^ means p < 0.05 and the difference was statistically significant.

### Relationship Between gOGTT (FPG, 1hPG, and 2hPG) and Postpartum Glucose

When studying the relationship between gOGTT (FPG, 1hPG, and 2hPG) and postpartum glucose status, patients with GDM in group A1 (<5.1 mmol/L), and B1 (gOGTT-1h PG <10.0 mmol/L) are more likely to have a normal postpartum oral glucose tolerance test (ppOGTT) result than those in group A2 (gOGTT-FPG ≧5.1 mmol/L) and B2 (gOGTT-1h PG ≧10.0 mmol/L), respectively, but this difference was not statistically significant (78/156, 83.0% vs. 49/156, 79.0%, *P* = 0.535, 62/156, 82.7% vs. 65/156, 80.2%, *P* = 0.936, Table 3). The rate of normal ppOGTT measurements in group C1 (gOGTT-2h PG <8.5 mmol/L) was higher than that in group C2 (gOGTT-2h PG ≥8.5 mmol/L), and this difference was statistically significant (61/156, 88.4% vs. 66/156, 75.9%, *P* = 0.046; Table 3).

When using ppOGTT (normal and abnormal) as the dependent variable and parameters, including age, nationality, education level, level of physical activity, menstrual regularity, history of macrosomia, history of GDM, family history of GDM, number of total pregnancies, labor duration, pre-pregnancy BMI, gOGTT-1h, gOGTT-2h, and weight gain, during pregnancy as independent variables, only gOGTT-2hPG entered into the equation in stepwise logistic analysis. The result indicated that an abnormal gOGTT-2hPG was a risk factor for abnormal ppOGTT (*P* = 0.04), and the risk of abnormal ppOGTT results in patients with GDM who had an elevated gOGTT-2hPG (≧8.5 mmol/L) was 2.426 times higher than that of patients with normal gOGTT-2hPG (<8.5 mmol/L; Table 5).

**Table 5:** The relationship between gOGTT-2hPG and ppOGTT (*n* = 156).

**Subject**	***b***	***S_**b**_***	***Wald x^**2**^***	***P***	***EXP(b)***
Constant	−2.031	0.376	29.186	<0.001	0.131
gOGTT-2h grouping	0.886	0.452	3.848	**0.040**	**2.426**

*GDM, gestational diabetes mellitus; gOGTT, gestational oral glucose tolerance test; 2h, 2 hours; PG, postprandial glucose; ppOGTT, postpartum oral glucose tolerance test. The bold values means p < 0.05 and the difference was statistically significant*.

### The Relationship Between Pre-Pregnancy BMI and Macrosomia

A Pearson's chi-square test indicated that the rate of macrosomia across the different pre-pregnancy BMI groups was not equivalent (*P* = 0.015). Further chi-square analysis with double segmentation showed that the rates of macrosomia in the normal and overweight group were higher than the underweight group (5.4 vs. 0.0%, 21.4 vs. 0.0%), but the difference was not statistically significant (*P* = 1.000, *P* = 0.069). In addition, the rate of macrosomia in the overweight group was higher than that of the normal weight group (21.4 vs. 5.4%), and the difference was statistically significant (*P* = 0.015), but the result may be affected by the small sample size (*n* < 60).

## Discussion

### It Is Important for Patients With GDM to Receive Guidance From a Professional Nutritionist to Manage Their Diets Effectively

Gestational diabetes mellitus is a common pregnancy complication, has been related to substantial short-term and long-term adverse health outcomes for both mothers and off springs (17). Identifying risk factors, in particular modifiable factors, is very important for preventing GDM among high-risk populations (18). Previous studies have reported that high-total fat and cholesterol and overall high-energy intake are significant risk factors for GDM, with total fat intake found to be significantly higher among women with GDM than in pregnant women who do not develop GDM (19). Our research showed that 73.7% of energy intake of patients with GDM was insufficient, with an improper proportion of carbohydrates and protein in the diet and an overly sufficient amount of fat. The result is inconsistent with what we traditionally think of as excess energy intake in people with GDM. The possible reason is that these patients controlled their own diet at home before they came to the nutrition department after being diagnosed with GDM in the obstetrics department. Zhang and Ning (20) found that replacing carbohydrates with fat was associated with a higher GDM risk. Therefore, it is important for patients with GDM to receive guidance from a professional nutritionist in order to manage their diets effectively.

### High Pre-Pregnancy BMI Was the Risk Factor for High OGTT-FPG Levels During Pregnancy

A number of known risk factors for GDM have become increasingly prevalent among pregnant Chinese women: the average age of pregnant women is increasing, the prevalence of overweight in Chinese adults has nearly tripled from 11.7 to 29.2% over the past two decades (21), according to Data From China National Nutrition and Health Survey in 2010–2012. Excessive body weight gain during pregnancy is an independent risk factor for GDM (22–26). Our study also found that high pre-pregnancy BMI was the risk factor for gOGTT-FPG levels during patients with GDM. Patients with GDM with a gOGTT-FPG ≧5.1 mmol/L had significantly higher pre-pregnancy BMI than those with a gOGTT-FPG <5.1 mmol/L. Furthermore, our study found that patients with GDM who were overweight before pregnancy were 4.583 times greater than patients with a normal weight before pregnancy to have a greater risk for a higher gOGTT-FPG (*P* = 0.021, OR = 4.583). In addition, this study also found that the rate of macrosomia in the overweight group was higher than that of the normal weight group. It is foreseeable that controlling pre-pregnancy BMI may help reduce the incidence of GDM and infant complications.

### Abnormal gOGTT-2hPG Result (≥8.5 mmol/L) Was a Risk Factor for an Abnormal ppOGTT Result

After delivery, all women with GDM must be re-evaluated by a 75 g OGTT (WHO criteria) 4–12 weeks postpartum to reclassify the glucose tolerance and every 2 years in cases of normal glucose tolerance. Abnormal glucose metabolism in postpartum patients with GDM is very common. Albareda reported that in the GDM patient group, the cumulative risk for diabetes and abnormal glucose tolerance was 56.2% (13.8% was diabetes, 42.4% was IFG or IGT) after 11 years (27). A standard treatment approach, such as dietary advice, self-monitoring of blood glucose levels, and insulin therapy, as needed could help reduce adverse perinatal outcomes for patients with GDM (28). The Australian Carbohydrate Intolerance Study in Pregnant Women (ACHOIS) and the Maternal-Fetal Medicine Units Network (MFMUN-GDM) clinical trials for the treatment of GDM showed that diagnosis and treatment of GDM improve pregnancy outcomes (29, 30). Tianjin GDM prevention plan found that abnormal postpartum glucose tolerance rates are 30.2% (diabetes 6.6%, IGT 10.1%, and IFG 13.5%) after treating with lifestyle intervention (31). All women with GDM should receive nutritional counseling, and increase physical activity according to the standards of Medical Care for Type 2 Diabetes in China 2019 (32). Our study found that 127 patients with GDM cases had normal blood glucose levels (81.4%), and abnormal postpartum glucose tolerance rates are 20.5%. Among them, 18.6% were pre-diabetic, and 1.9% were diabetes. The results may due to that we used WeChat (a cell phone APP popular used in China) to manage patients with GDM, but we still need more prospective studies with a large sample size of the patients with GDM to verify our result.

Many studies have found that elevated fasting glucose during pregnancy was a risk factor related to abnormal ppOGTT (33–35), while there are few studies on the relationship between 1- and 2-h glucose levels of gOGTT and the outcome of postpartum OGTT. Our results showed that abnormal gOGTT-FPG and gOGTT-1hPG results did not affect the maternal postpartum blood glucose outcomes. However, an abnormal gOGTT-2hPG result (≥ 8.5 mmol/L) was a risk factor for an abnormal ppOGTT result. Patients who had an elevated gOGTT-2hPG (≥8.5 mmol/L) were at 2.426 times greater risk of an abnormal ppOGTT when compared to patients with normal 2 h glucose levels (<8.5 mmol/L). The reason may be the different judgment thresholds of OGTT during pregnancy and postpartum. The normal value of fasting glucose of gOGTT and ppOGTT is <5.1 and 6.1 mmol/L, respectively; while the normal value of 2 h glucose of gOGTT and ppOGTT is <8.5 and 7.8 mmol/L, respectively.

### Limitations of the Study

The limitations of our study are the relatively heterogeneous population and because of the small number of patients, these findings need to be corroborated in larger cohorts. However, we will enlarge the samples in the following study and extend the follow-up to 1, 5, and 10 years. In the future, more prospective studies with a large sample size are needed to confirm our findings.

## Conclusion

Gestational diabetes mellitus patients usually have insufficient energy intake, the improper proportion of carbohydrates, protein, and fat, which makes it important for them to receive guidance from a professional nutritionist. We have identified some of the markers as predictors of gestational diabetes and postpartum abnormal glucose tolerance following GDM: pre-pregnancy obesity, high level of gOGTT-2 h-postprandial glucose (gOGTT-2h PG). On the basis of these results, close obstetric and metabolic follow-up during pregnancy and regular follow-up of GDM patients at greater risk of developing diabetes after pregnancy must be reinforced, if we are to prevent diabetes in these women.

## Data Availability Statement

The original contributions presented in the study are included in the article/supplementary material, further inquiries can be directed to the corresponding author/s.

## Ethics Statement

The studies involving human participants were reviewed and approved by Institutional Review Boards of the Third Xiangya Hospital, Central South University (No. 2020S262). The patients/participants provided their written informed consent to participate in this study.

## Author Contributions

CM and ML designed the study and were the guarantors of this work. HoL conducted, analyzed, interpreted the data, and wrote the manuscript. HP, CC, HuL, MW, GF, HN, and JW performed the data collection. All authors approved the final version of this manuscript.

## Funding

This work was supported by the Scientific Research Project of Hunan Provincial Health Commission [no. B2017032] and the Program of Science and Technology Plan of Hunan Provincial Science and Technology Department [no. 2011FJ3254].

## Conflict of Interest

The authors declare that the research was conducted in the absence of any commercial or financial relationships that could be construed as a potential conflict of interest.

## Publisher's Note

All claims expressed in this article are solely those of the authors and do not necessarily represent those of their affiliated organizations, or those of the publisher, the editors and the reviewers. Any product that may be evaluated in this article, or claim that may be made by its manufacturer, is not guaranteed or endorsed by the publisher.
